# Triple-Negative Lobular Breast Cancer Causing Hydronephrosis

**DOI:** 10.1177/2324709620905954

**Published:** 2020-02-11

**Authors:** Michael Chahin, Hardik Chhatrala, Nithya Krishnan, Darren Brow, Lara Zuberi

**Affiliations:** 1 University of Florida, Jacksonville, FL, USA

**Keywords:** breast cancer, receptor, negative, triple, hydronephrosis

## Abstract

Breast cancer is the leading malignancy and the second most common cause of mortality in women. Although there have been advances in identifying biomarkers as potential targets for therapy, triple-negative breast cancer (TNBC) continues to have a poorer prognosis than the other receptor subtypes. The most common sites of metastasis are bone, liver, lung, and brain. We present a patient with known TNBC presenting with nausea and vomiting in whom computed tomography revealed a right-side pelvic mass causing hydronephrosis. Biopsy was consistent with TNBC of the ureter, an unusual site for breast cancer involvement. She required ureteral stent placement to relieve obstruction and has had good response to paclitaxel. Hydronephrosis due to malignancy presents significant risk of morbidity and mortality due to compromised renal function and must be resolved promptly to avoid compromise of renal function.

## Introduction

Approximately 15% of breast cancers are triple negative, in which the tumor lacks expression of estrogen receptor (ER), progesterone receptor (PR), and human epidermal growth factor receptor 2 (HER2).^1^ However, the representation of triple-negative subgroup is much higher in those that recur or metastasize (5-year overall survival is 77% for triple negative vs >90% for others). Breast cancer most commonly metastasizes to bone, liver, lung, and brain.^2^ A Surveillance, Epidemiology, and End Results database–based study demonstrated triple-negative breast cancer (TNBC) has a propensity to metastasize to bone.^3^ In recent years, TNBC has been recognized as comprising a heterogeneous group of tumor types. This has revealed different potential biomarkers that may be treated with targeted therapy. In general, however, TNBC is an aggressive entity that requires prompt recognition and treatment.^4^ We present a patient with TNBC who developed bulky pelvic lymphadenopathy with subsequent invasion of the right ureter.

## Case Report

A 54-year-old female with a history of triple-negative grade 1 ductal adenocarcinoma diagnosed 2 years prior presented to the emergency department with diffuse abdominal pain, nausea, and vomiting that had been worsening over 2 weeks. She had pursued breast cancer treatment with holistic and natural remedies only. Breast examination revealed a 10-cm right inferior breast mass and peau d’orange changes. There was bilateral axillary lymphadenopathy.

**Figure 1.**
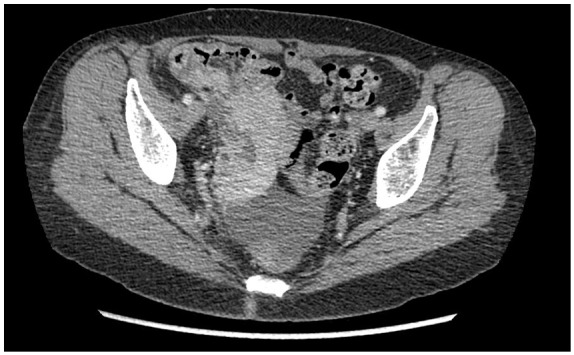
Right hemi-pelvis bulky mass.

**Figure 2.**
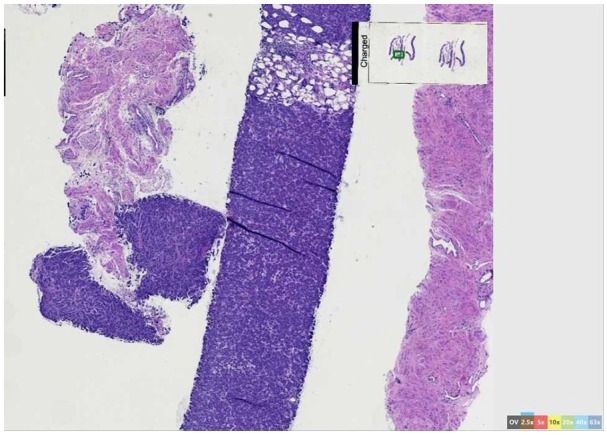
Metastatic poorly differentiated adenocarcinoma of the breast. Nearly entire biopsy is tumor cells.

**Figure 3.**
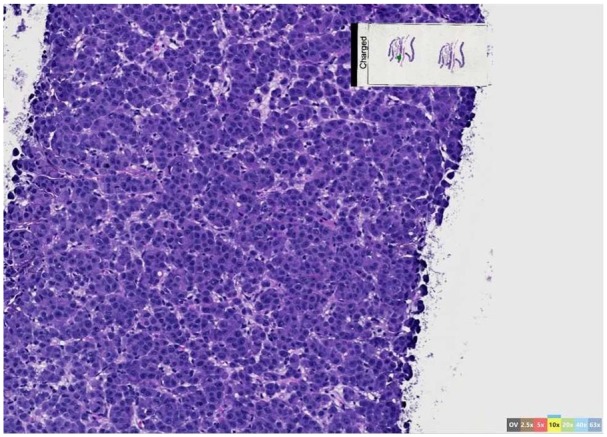
Tumor cells at 20× with high degree of atypia and poor differentiation, obtained from right pelvic mass.

**Figure 4.**
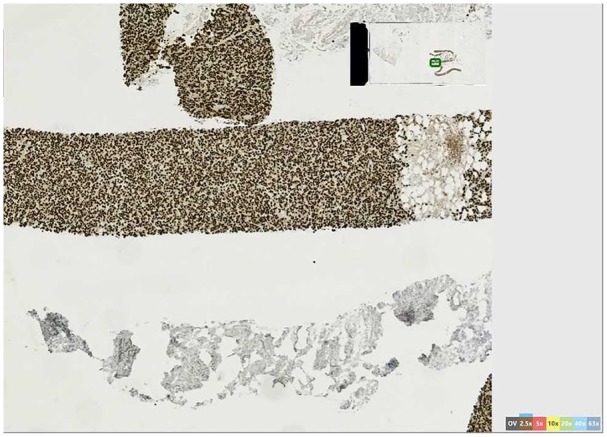
Gata3 immunostaining demonstrating epithelial tissue, 96% positive in metastatic breast cancer.

Computed tomography scan showed right distal ureteral thickening and right hemi-pelvic mass causing severe hydronephrosis (Figure 1). Biopsy of the right pelvic mass revealed poorly differentiated adenocarcinoma that was GATA3, CKAE1/AE3, and cytokeratin (CK) 8/18 positive, consistent with breast primary tumor (Figures 2-4). The sample demonstrated smooth muscle indicating ureteral or bladder invasion. The mass was ER negative, PR negative, HER2 negative, and Ki-67 positive. A core needle biopsy of left breast mass from the outside hospital 2 years prior revealed invasive pleomorphic lobular carcinoma Nottingham grade 1, ER, PR, and HER2 negative.

She underwent a right ureteral stent placed to relieve the obstruction. She received whole brain radiation therapy for cerebellar metastases that were found on brain imaging. Positron emission tomography scan a few weeks after this presentation revealed increased metabolic activity of bilateral breasts and axillary, retroperitoneal, mediastinal, cervical, and supraclavicular lymph nodes. The right pelvic mass was demonstrated again, and an omental mass was present. Bone or liver metastasis were not evidenced though there were hypermetabolic lower lobe pulmonary opacities bilaterally. Palliative chemotherapy was started in the form of single-agent weekly paclitaxel. She has had excellent response to whole brain radiotherapy with decrease in tumor size, and no neurologic deficits. Her clinical course was complicated by pseudomonas pyelonephritis after cycle one but has otherwise tolerated 3 cycles of paclitaxel well.

## Discussion

Information regarding ureteral involvement by breast cancer is limited. The available studies consist largely of individual case reports and case series. An autopsy review of 215 patients with breast malignancy revealed 42 cases of ureteral metastasis.^5^ A case series of 82 patients demonstrated cervical, prostate, breast, and colorectal as the most common primary cancers with ureteral metastasis.^6^ Lymphoma involvement of the ureter has also been reported.^7^ Considering these autopsy reports, it is likely that ureteral metastasis is underrepresented. A limit to these studies is that they predate widespread use of computed tomography scan, but they provide evidence for ureteral metastasis from breast cancer as an entity.

Metastasis of breast cancer to the ureter is unusual, though more likely to occur from breast cancer than other cancer types simply by virtue of its high incidence. It has been suggested that hematogenous or lymphatic routes of metastasis occur less frequently than direct invasion. This is likely due to the separate vasculature between the 3 anatomic portions of the ureter.^8,9^ Our patient likely had distal right ureteral invasion from the adjacent pelvic mass, possible extracapsular, as opposed to hematogenous or direct lymphatic spread. Anatomically, the likelihood of breast cancer developing bulky lymphadenopathy is low, but given our patient’s extensive lymphatic involvement, we propose that the pelvic mass extended from a regional lymph node though no lymph node tissue was isolated in the biopsy. This is opposed to a case reported by Gabsi et al, in which breast tumor cells were found within the ureter with no regional lymph node metastasis.^10^

Based on our literature review this is the only case demonstrating TNBC with invasion of the ureter. ER+/PR−; HER2+, and ER+/PR+; HER2+ breast malignancies have been reported.^11^ One explanation for the rarity of this is that the aggressive nature of most TNBCs often results in multiple sites of metastases that are more accessible for biopsy. This was more a case of a neglected cancer rather than an inherently aggressively behaving cancer given that it was low-grade and the patient had elected not to pursue treatment for more than 2 years.

The management of patients of malignant urinary tract obstruction is largely palliative. Placement of retrograde ureteral stents or percutaneous nephrostomy have been employed.^12^ In addition to addressing the morbidity of renal obstruction, these patients should be treated with the appropriate systemic therapy depending on receptor status and performance status. In solid tumors, ureteral obstruction whether through direct invasion or extrinsic compression is often seen as a surrogate for advanced cancer. Fortunately, some common chemotherapy agent classes used in the palliative setting for metastatic breast cancers like taxanes, anthracyclines, and eribulin have minimal renal excretion.^13^ They can be used safely in these subgroups of patients with obstructive uropathy secondary to ureteral obstruction.

## Conclusion

Hydronephrosis is a rare but significant complication in cancer patients. It poses risk of worsening morbidity including the need for dialysis and the higher anticipated toxicity of chemotherapeutic agents and increases the therapeutic challenges as in our patient who had a pseudomonas urinary tract infection. It needs to be addressed promptly by the treating oncologist in collaboration with a multidisciplinary team of urologists and radiologists.
